# What Are the Research Priorities for the Dyslexia Community in the United Kingdom and How Do They Align With Previous Research Funding?

**DOI:** 10.1002/dys.70004

**Published:** 2025-03-18

**Authors:** Catherine Manning, Beverley Jennings, Keren MacLennan, Raveen Rayat, Keith Spiller, Holly Joseph

**Affiliations:** ^1^ School of Psychology University of Birmingham Birmingham UK; ^2^ School of Psychology and Clinical Language Sciences University of Reading Reading UK; ^3^ Centre for Developmental Science University of Birmingham Birmingham UK; ^4^ Institute of Education, University of Reading Reading UK; ^5^ Department of Psychology University of Durham Durham UK; ^6^ Department of Psychology University of Bath Bath UK; ^7^ Department of Sociology, Social Policy and Criminology University of Southampton Southampton UK

## Abstract

Targeting research towards areas that matter to dyslexic people and their families is essential for improving dyslexic people's lives. However, we do not know what the dyslexia community's research priorities are and whether they align with current research funding. We categorised previous funding for dyslexia research in the United Kingdom and considered how this aligns with community research priorities, using multiple methods and a participatory approach. We identified that the vast majority (78%) of funding has been spent on research into biology, brain and cognition. Through seven focus groups (*n* = 37), dyslexic adults and/or family members/carers of dyslexic children expressed that this balance needs redressing, and we identified four important areas for future research, informed by lived experiences. Finally, 436 members of the dyslexia community ranked the importance of research topics in a survey. The top five priorities related to *training teachers and professionals*, *educational supports and interventions*, *mental health and self‐esteem*, *making services and spaces more inclusive* and *cognition*. Research into genes and risk factors was less prioritised. These results provide a point of reference for researchers and funders to align future research funding with the dyslexia community's priorities so that it can be of translational benefit.

**Summary**

- Most funding for UK dyslexia research has been awarded to projects studying Biology, brain and cognition.
- Dyslexia community members felt that more research funding should go to other research areas, including those with clear practical relevance.
- A survey revealed the community's top priorities were teacher training, educational supports, mental health and self‐esteem, improving inclusion and cognition.
- Community members perceived the study of genes and risk factors to be less important than other research areas.
- Researchers and funders should align their own priorities with those of the dyslexia community to ensure research can benefit stakeholders.

## 1 Introduction

Historically, research funders have set their agendas with little involvement of community members (Cartier et al. 2018). Consequently, research is often misaligned with community priorities (Chalmers et al. 2014; Crowe et al. 2015). However, it is being increasingly recognised that community involvement is necessary for ethical research and can help improve health outcomes, build public trust in research (Solomon et al. 2016) and minimise research waste (Chalmers and Glasziou 2009). Accordingly, funders are increasingly incorporating community perspectives into their strategies and funding decisions (NIHR 2015).

However, no studies have yet characterised previous research funding for developmental dyslexia and asked dyslexic people and their families (henceforth, the dyslexia community) what they would like to be researched, so that we do not know if this mismatch between research funding and community priorities is also occurring in dyslexia research. Dyslexia is under‐researched and receives substantially less research funding than less common developmental conditions like autism (Bishop 2010). It is therefore critical to identify any mismatch between research funding and community priorities so that we know where to target these relatively limited resources in future to ensure benefit to the community.

A discrepancy between research conducted and community priorities has already been established in autism research. Pellicano et al. (2013) quantified the United Kingdom's (UK) autism research grant funding awarded to each of the categories set out by the United States (US) Interagency Autism Coordinating Committee (IACC 2009, 2010). They reported that the majority of funding went to projects investigating Biology, brain and cognition (56%), with far less spent on research into Interventions (18%), Causes (15%), Diagnosis (5%), Services (5%) and Societal issues (1%). Other portfolio analyses of autism research funding have also shown a predominance of funding into basic, biomedical science in the US (Harris et al. 2021; Singh et al. 2009), Canada (Krahn and Fenton 2012) and Australia (den Houting and Pellicano 2019). Focus groups, interviews and an online survey with autistic adults, parents of autistic children, practitioners and autism researchers revealed that the distribution of funding reported by Pellicano et al. (2013) did not reflect community priorities (Pellicano et al. 2014). Many participants commented that funding should be more evenly distributed across categories, with less ‘basic science’ and more research into matters affecting everyday life (see also Cage et al. 2024). A similar redistribution of emphasis is desired by community stakeholders in UK research into genetic syndromes (Down Syndrome, Fragile X syndrome and Williams Syndrome; Cristescu et al. 2024).

Here, we aimed to understand whether there is a similar mismatch between funding and community priorities in UK dyslexia research. While there is an established autism community and the importance of including autistic people in research is widely acknowledged (though not always actioned; Keating 2021), this is much less the case for dyslexia. Without a strong community voice, it is especially important that we gain the views of those with dyslexia and their families. Existing studies have identified community priorities for neurodevelopmental and/or learning disabilities, but not dyslexia specifically. The British Academy of Childhood Disability James Lind Alliance partnership (Morris et al. 2015) identified research priorities for ‘neurodisability’: a term encompassing a range of conditions affecting the nervous system, including cerebral palsy, autism and epilepsy. The most highly prioritised research questions related to the effectiveness of therapies, improving communication, and strategies to improve inclusion and participation. However, it was unclear if members of the dyslexia community were involved. A James Lind Alliance partnership for Developmental Language Disorder identified similar priorities, including those relating to interventions, outcomes, and teacher training (Kulkarni et al. 2022). Particularly relevant for the current study is a James Lind Alliance partnership that established research priorities for children and young people with learning difficulties, including dyslexia, in Scotland (Lim et al. 2019). The top priorities included identifying the knowledge and training education professionals need to identify learning difficulties early and provide support, understanding optimal educational and community environments, and understanding how various professionals and parents can best work together.

However, it is important to understand the specific issues that face members of the dyslexia community and how this affects their priorities for research: we cannot assume that the dyslexia community will have the same priorities as communities relating to other learning difficulties. Dyslexia research, perhaps more so than with other neurodevelopmental conditions, has been plagued with controversies ranging from whether it exists (Elliott 2020), what causes it (Stein 2018) and what will help dyslexic readers (Nicolson et al. 2001), leading to widespread belief in interventions without a strong evidence base (e.g., coloured overlays; Griffiths et al. 2016). These specificities in dyslexia research could lead to distinct community views about research priorities that are not shared by communities associated with other learning difficulties. Likewise, the funding allocated to dyslexia research projects could look quite different to that allocated to other conditions. Research is typically siloed according to different developmental conditions (Astle and Fletcher‐Watson 2020) and because dyslexia is typically identified by education rather than health professionals, it may fit less well with the remit of medical funders than conditions like autism and ADHD. It is therefore important to provide dyslexia researchers and funders of dyslexia research with tailored insights into the relevant community's perspectives.

While there are a range of valid approaches to research priority setting (Nasser et al. 2013; Viergever et al. 2010), in this study, we particularly wanted to foreground the lived experiences and perspectives of dyslexic people and their families and carers. In Study 1, we categorised grant expenditure for UK dyslexia research. In Study 2, we used focus groups to understand community members' reactions to this funding allocation and to encourage them to reflect on their lived experiences to develop target areas for future research. In Study 3, we used these insights to inform a survey where we quantified research priorities. We used participatory research practices (Cornwall and Jewkes 1995) to inform the design and conduct of the project. Specifically, our research questions were:

1. How is UK dyslexia research funding allocated across different research areas? (Study 1)
2. What do the dyslexia community think about this funding allocation? (Study 2)
3. What areas are important to the dyslexia community, and which lived experiences have shaped these? (Study 2)
4. Which research topics are most and least prioritised by the dyslexia community? (Study 3)

## 2 Study 1. Categorising Grant Expenditure for Dyslexia Research

### 2.1 Methods

We searched for UK grants awarded between 1999 and March 2022 in Europe PMC's Grant Finder and UKRI's Gateway to Research, searching for the keywords ‘dyslexi*’ (or dyslexic OR dyslexia), ‘reading disorder’, ‘reading disability’, ‘reading deficit’, ‘reading difficulty’, ‘reading difficulties’, ‘literacy disorder’, ‘literacy difficulty’, ‘literacy difficulties’, ‘struggling readers’, ‘poor readers’, ‘reading impairment’ or ‘impaired readers’ in the title or abstract. We then checked the websites and annual reports of individual funders, including the Wellcome Trust, Leverhulme Trust, Baily‐Thomas Charitable Fund, Nuffield Foundation, British Academy and National Institutes for Health Research. Finally, we ran a Scopus search for UK publications in the journal ‘Dyslexia’ and extracted funding information to identify any missed grants. We collated projects across sources and removed duplicates. We (C.M., H.J. and R.R.) collectively decided to exclude grants which fund a research centre rather than a defined project (*n* = 5) because these were difficult to categorise given the broad research aims, with dyslexia often only relating to a small part of these aims, and grants which we agreed were not relevant to developmental dyslexia (e.g., studies mentioning dyslexia only as a possible follow‐up study or implication; studies of acquired dyslexia; *n* = 59), resulting in 60 projects. We included projects which included participants with reading disorders, even if not specifically diagnosed with dyslexia, as we reasoned that there could be unidentified dyslexic individuals in these samples and that excluding these studies might cause certain research categories to be under‐represented (e.g., studies of societal issues and services might be less concerned with the presence/absence of a diagnosis). However, the same pattern of funding allocation was found with these projects excluded. We excluded projects looking at the normal distribution of reading across the population so that we could relate the funding distribution to the priorities of the dyslexia community. We contacted Principal Investigators and funders to complete missing information (e.g., abstract or amount awarded). In 13 cases, we did not obtain the full abstract but collated other information (e.g., impact summaries, published outcomes, publications linked to the grant and author websites). The amount awarded for some studentships was missing, so we estimated these as the average of other studentships (£84,635).

We categorised each project according to the six categories and 35 subcategories used by the IACC's (2009) portfolio analysis of US autism research and Pellicano et al. (2013), enabling comparisons with similar analyses. The six main categories were: (i) Diagnosis, characteristics and behaviour; (ii) Biology, brain and cognition; (iii) Causes; (iv) Support and interventions; (v) Services and (vi) Societal issues. We made minor wording edits for some categories to better reflect dyslexia research (e.g., ‘characteristics’, rather than ‘symptoms’). The categorisation was initially carried out independently by two psychology undergraduate researchers, and then a consensus was reached with senior researchers (C.M. and H.J.). In cases where the project spanned multiple (sub)categories, we chose the subcategory that reflected the predominant emphasis of the project. Our spreadsheet of identified projects and coding scheme is at https://osf.io/sgy7t/.

### 2.2 Results

Of the 60 research projects identified, 34 (56.7%) were categorised as relating to Biology, brain and cognition; 11 (18.3%) for Support and interventions; 7 (11.7%) for Diagnosis, characteristics and behaviour; 5 (8.3%) for Causes; 2 (3.3%) for Services; and 1 for Societal issues (1.7%). The total amount awarded was £15,942,383.

**FIGURE 1:**
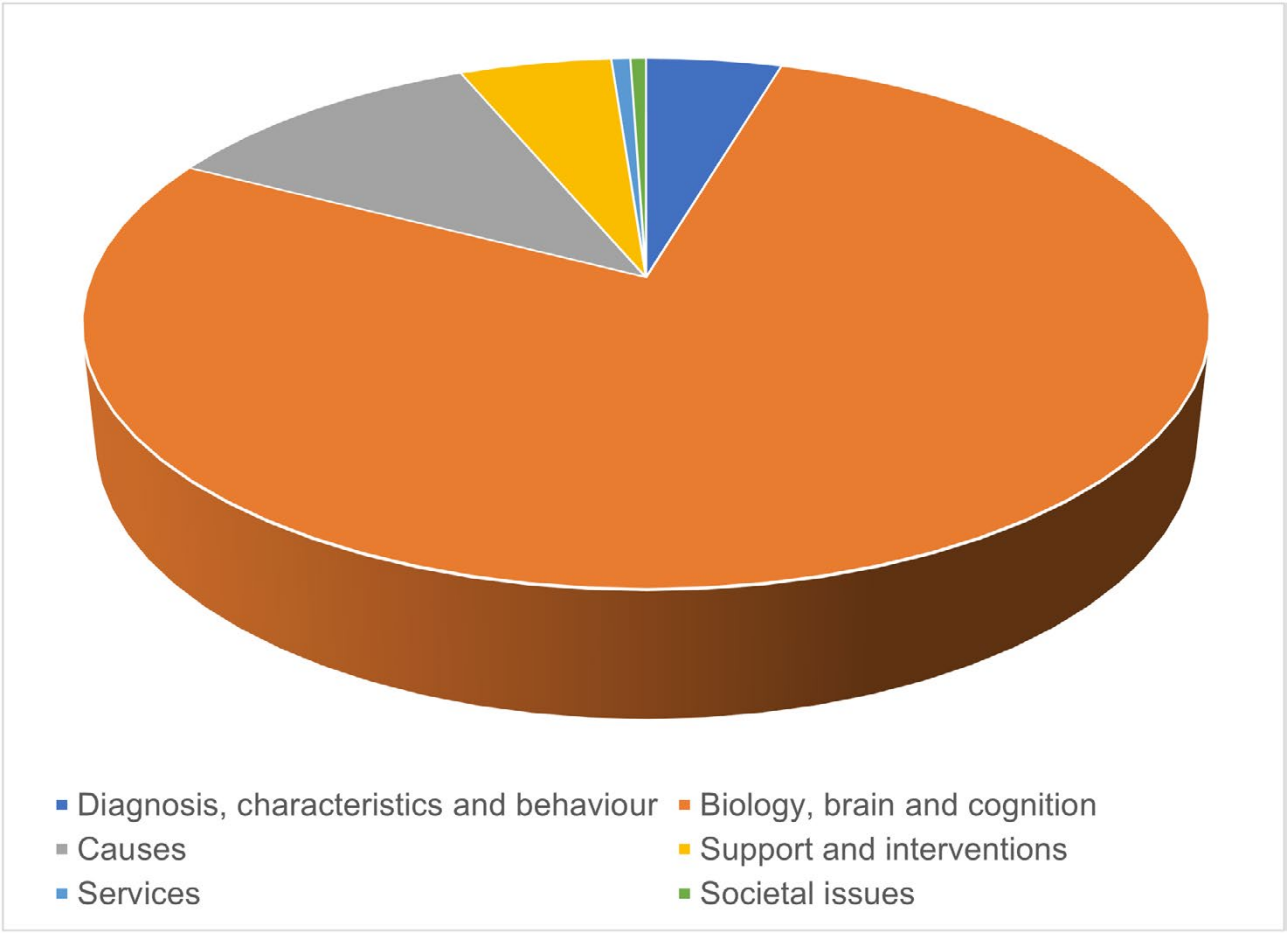
Amount awarded to projects within each research area category as a proportion of total spend (~£15.9 million).

As Figure 1 shows, the vast majority was awarded to Biology, brain and cognition projects (£12,434,219; 78.0%), with far less awarded to Causes (£1,765,660; 11.1%); Support and interventions (£822,440; 5.2%); Diagnosis, characteristics and behaviour (£732,595; 4.6%); Services (£102,835; 0.6%) and Societal issues (£84,635; 0.5%). Within the 34 projects in the Biology, brain and cognition category, 16 were in the subcategory ‘Cognitive studies’, collectively receiving £2,357,696. While there were only six projects in the subcategory for ‘Sensory and motor function’ and four for ‘Neural systems’, these were more costly, receiving £3,699,944 and £2,598,646, respectively (Table S1).

### 2.3 Summary

As in UK autism research (Pellicano et al. 2013), we found a striking predominance of funding for Biology, brain and cognition in dyslexia research. Meanwhile, more applied topics like Services, Societal issues, Support and interventions received scant funding. In Study 2, we ask whether this funding distribution aligns with the community's priorities.

## 3 Study 2. Focus Groups With the Dyslexia Community

### 3.1 Methods

#### 3.1.1 Focus Groups

Three semi‐structured focus groups for dyslexic adults and four groups for parents/carers and immediate family members of dyslexic people were conducted online over Microsoft Teams. The focus group schedule (https://osf.io/sgy7t/) was developed in consultation with community members. We aimed to ensure our schedule was inclusive of those who were unfamiliar with academic research by first asking participants to answer general questions about what dyslexia means to them and the challenges associated with dyslexia. The aim here was to establish rapport and encourage participants to reflect on their experiences relating to important issues for them, as we did not expect participants without a research background to be able to formulate research questions independently. Next, we gave a short presentation on our preliminary analysis showing what dyslexia research has previously been funded to capture participants' reactions to this funding distribution and whether it matched their priorities (Pellicano et al. 2014). The presentation began with an introduction to funding processes and example projects belonging to each funding category to provide participants with the knowledge needed to participate in this discussion (Pratt 2021), and participants had the chance to ask questions. Finally, we asked participants about the most important questions to be addressed by future dyslexia research.

The schedule was sent to participants before sessions, with a code of conduct. The focus groups for dyslexic adults were facilitated by a dyslexic researcher, and the groups for parents/carers/family members were facilitated by a researcher who is a parent/carer of a dyslexic child. The facilitator ensured that all participants were included in the discussion. An additional researcher assisted with technological issues and note‐taking. The University of Reading School of Psychology and Clinical Language Sciences Research Ethics Committee (SREC) provided approval (2021‐190‐CM), and all participants provided informed consent.

#### 3.1.2 Participants

Participants were required to be adults (18+ years) living in the UK who were dyslexic themselves and/or parents/carers/immediate family members of someone with dyslexia. Participants were recruited through charities, social media and research databases. Following advice from community organisations, we included participants who self‐identify as dyslexic to strive for a more representative sample, as certain parts of the population, including those with lower socioeconomic status, are less likely to receive a diagnosis (Knight and Crick 2021; Siegel and Himel 1998). However, all participating dyslexic adults had a diagnosis. All family members were parents/carers. Thirty‐seven participants (see Table 1 for demographics) took part across the groups for dyslexic adults (*n* = 5, *n* = 7 and *n* = 3) and parents/carers (*n* = 7, *n* = 5, *n* = 6 and *n* = 4). Five participants were both dyslexic adults and parents/carers of dyslexic children, with one choosing to join a parent/carer focus group and four choosing to join a dyslexic adult focus group. Three participants reported an additional role in the dyslexia community (teacher, specialist teacher and dyslexia assessor).

**TABLE 1:** Frequencies of focus group participant demographics.

Response	Frequency (total *n* = 37)
Age range	
18–24	4
25–34	3
35–44	9
45–54	19
55–64	2
Gender	
Female	32
Male	5
Ethnic group	
White British	28
White—Other	4
Black Caribbean	2
Indian	1
Mixed	2
Highest level of education	
Higher degree	12
Degree	17
A‐levels/equivalent	5
Vocational/other	3
Read or hear about dyslexia research	
1—Never	6
2	13
3	10
4	7
5—Regularly	1
Previous participation in dyslexia research	
Never	32
Less than once every 2–3 years	1
Once every 2–3 years	2
Once a year	2

*Note:* Frequencies are provided across all focus groups and not split up according to parents/carers and dyslexic adults to help preserve the anonymity of participants.

#### 3.1.3 Analysis

The data were transcribed and checked before analysis. To understand community reactions to previous funding, C.M. (a non‐dyslexic dyslexia researcher) immersed herself in the data before using qualitative content analysis with an inductive, iterative process (Elo and Kyngäs 2008; Selvi 2019) to develop a coding frame for categories and subcategories relating to ‘what people think about previous funded research’. Using NVivo 14 (Lumivero 2023), C.M. used open coding (making initial notes), then generated lists of categories which she grouped into higher‐order categories. C.M. defined and revised these (sub)categories to avoid overlap and then coded the whole data set.

To understand the community's priorities and the experiences that have shaped them, K.M. used reflexive thematic analysis (RTA; Braun and Clarke 2019), to develop themes and subthemes reflexively and iteratively using NVivo 10 (Lumivero 2014). K.M. analysed the whole data set, as important lived experiences were discussed both before and after the funding presentation. As participants were not researchers themselves, K.M. developed priority areas for future research from participants' experiences, even when these were not formulated as a research question by participants. RTA recognises the researcher's role in developing meaning and interpretation and the subjectivity of participants and researchers. K.M. is active in neurodiversity research but has less dyslexia‐specific research expertise and no lived experience.

First, K.M. familiarised themselves with the data by reading the transcripts and noting initial impressions. Second, they iteratively coded the data set, considering the meaning being communicated by participants and the interpretation of this meaning in relation to the research aims. During this stage, K.M. began to get a sense of some themes and examined the codes to develop initial themes that represented broader meanings. They examined the viability of these themes and re‐engaged with the codes and data set to review and refine themes so they reflected meaningful concepts and ideas. Finally, K.M. refined the theme names that represented these concepts. Throughout this process, they reflected on where they had applied their own meaning or prior knowledge to the analysis to try to maintain a data‐driven approach. However, K.M.'s prior knowledge and experience of neurodiversity, alongside the research aims, shaped interpretations of participants' meanings.

### 3.2 Results

#### 3.2.1 Community Perspectives on Previous Funding

In the final coding frame for the content analysis, there were categories for Emotional Responses, Views on Funding Allocation across Research Categories, Perceived Reasons for Funding Allocation and Views on Funded Research (Table 2). Regarding emotional responses, five were not surprised about the focus on Biology, brain and cognition relative to other areas, whereas seven found it surprising or shocking. Two participants mentioned that all research areas were important, and seven said that research into Biology, brain and cognition was necessary. However, 11 participants said there was too much focus on medical research, including Biology, brain and cognition and Causes, and 19 said there was too little research on areas directly impacting people's daily lives. Here is an illustrative quote:

… 12.7 million to the uh biology, brain and cognition… that's a huge percentage, that's kind of shocked me a bit. And seeing how little is spent on the services and the society issues and the support and intervention side of things. FG52, dyslexic adult

**TABLE 2:** Categories and subcategories of focus group participants' views on previously funded research.

Subcategory	*N* focus groups	*N* participants
Emotional responses		
Angry or frustrated	1	2
Disappointed	1	1
Horrified	1	1
Not surprised	4	5
Sad	1	1
Surprised or shocked	5	7
Views on funding allocation across research categories		
All research categories are important	2	2
Necessity of research into Biology, Brain and Cognition	5	7
Limited funding for areas which directly impact people's lives	7	19
Too much medical focus (Biology, Brain and Cognition and Causes)	7	11
Perceived Reasons for Funding Allocation		
Biology, Brain and Cognition research is expensive	1	1
Disconnect between what researchers/funders want and community need	3	3
Research into support is not profitable	1	1
Societal issues harder to research	1	1
Views on funded research		
Useful insights gained from research	2	4
Money spent has not been beneficial for community	5	7
Deficit‐focused	3	4
Focused on long‐term insights rather than supporting people now	2	2
Dyslexia community need to be leading the research	1	1
People with dyslexia and their families not the focus of the research	4	5
Research not accessible or translated	6	7

*Note:* N focus groups refer to the number of focus groups in which each subcategory was mentioned.

Participants suggested a few reasons for the presented funding allocation, including a disconnect between what researchers/funders want and what the community need (*n* = 3). Regarding views on previously funded research, four participants mentioned useful insights gained from previous research, including the understanding that dyslexia has a genetic basis; however, seven participants commented that the research had provided poor value for money, without leading to clear benefits to the community. Four participants commented that previous research (particularly brain‐based research) had been deficit‐focused, which can contribute to stigma. Seven participants also commented on previous research being inaccessible and not properly communicated and translated for the benefit of the dyslexia community.

#### 3.2.2 Priority Areas for Future Research

Four main themes (one with two subthemes) were developed relating to priority areas shaped by lived experiences (Table 3).

**TABLE 3:** Summary of the main and subthemes developed using reflexive thematic analysis relating to community priorities.

Main themes	Subthemes (where applicable)
Early, effective and accessible identification and diagnosis of dyslexia	
Effective support for dyslexic people and their families	
Improving understanding of and attitudes towards dyslexia	Recognising and valuing strengths as well as differences Understanding intersectional identities and social inequalities
Improving mental health and self‐esteem of dyslexic people	

##### 3.2.2.1 Early, Effective and Accessible Identification and Diagnosis of Dyslexia

Many participants described challenging journeys to accessing a diagnostic assessment, which seemed to be essential for accessing beneficial educational support. Many participants described barriers to accessing diagnostic assessment, meaning that diagnosis and the support that was then provided often came too late, already exposing children to avoidable and harmful challenges in education settings.

My main experience or issues with being dyslexic has all stemmed from when I was younger, so definitely… trying to sort of identify it earlier on. FG05, dyslexic adult

Several participants described experiences of delayed diagnosis due to screening tools that did not detect dyslexia in themselves or their children, which they perceived was due to tools not being sensitive enough to different presentations of dyslexia. Some participants described that the screening tools suggested they were not dyslexic, despite they themselves recognising they were dyslexic, and later receiving a diagnosis of dyslexia. It was perceived that this might be because school staff struggle to interpret the complexity of certain profiles in screening tool results.

My daughter was screened for the kind of year two screening dyslexia thing, and that threw up nothing. However, on later reflection, erm it was misread, so although it didn't, it did flag up that she has no signs of dyslexia, there were, um, her single word reading did flag up, although it wasn't under that heading in the report. So, I think even though the school had tried to do something, they then gave me the wrong impression… FG38, parent of dyslexic child

Due to the barriers to getting a diagnosis, those who had the financial means would pursue a private assessment. Some recognised their privilege to be able to do this and were concerned that other children might not have the same opportunity. They wondered about the detrimental impact this lack of diagnosis and support would have on children's future outcomes and prospects.

We do need early diagnosis, but we need affordable and accessible diagnosis because right now it's a lottery. If you happen to have parents who can afford it, you get the diagnosis. If you don't, you don't, and that's not right. FG07, parent of dyslexic child

##### 3.2.2.2 Effective Support for Dyslexic People and Their Families

Most participants expressed the importance of effective support and that often the support needs of individual children are not being recognised. Participants described how the lack of support limits opportunities to flourish in education and achieve their full potential. It was expressed that even for children who can maintain or achieve standards expected by schools, this is often short of a child's own potential and aptitude. Some reported that literacy challenges led to them being brought down into lower ability sets across multiple subjects, despite strengths in some subjects.

I was actually particularly good at maths. But because my English was so bad, they moved me to the same maths group as English. FG30, dyslexic adult and parent of dyslexic children

Some participants described that because dyslexic children are not getting the support they need in school, this places the responsibility on families, who could recognise the challenges their children experience and what could help in schools. Several parents could understand their child's challenges due to their own similar traits, and some described how they did their own research so they could understand more about dyslexia and support their child, seek diagnosis, and get external support. Many parents conveyed experiences of not being believed by schools and having to battle for their child's support needs to be recognised. This could be bolstered by negative screening results, and the eventual feeling of vindication when their child received a diagnosis. Parents with multiple dyslexic children portrayed how the first child's diagnostic journey armed them with knowledge, experience, and confidence to advocate for their other children.

Probably some of us have challenged the school and said if you're telling me my child isn't meeting expectations, can you explain why, you know I'm noticing this at home and that at home and it perhaps being downplayed by the school.… To the point where it can feel… quite confrontational and some people at that point may decide just to back off. I feel like there needs to be some support, not only for the child but also for the, the families behind them. FG13, parent of dyslexic children

Parents also described feeling pressure to ensure their dyslexic children achieve in school, with a few fearing that their child could be ‘kicked out’ if they did not maintain attainment. Parents conveyed that they have been expected to provide extra educational support for their children at home, which can strain relationships between parents and children. This, alongside parents' awareness that their children are not happy or flourishing in school, was viewed as having emotional consequences across family members.

…dyslexics are used to quite a lot of change. They're used to quite a lot of failure, well we are anyway in our family… So erm I think there is an emotional part of this… we have a lot of ups and downs and how that affects us in our life… FG03, dyslexic adult

Some dyslexic adults described experiences of feeling unsupported in workplaces and that current workplace assessments seemed unhelpful and resulted in inadequate support. A few conveyed being in careers where they could play to their strengths, but there were also experiences of having to struggle through certain job requirements without accommodations. A few described how career progression could be unfairly limited due to these challenges, and some parents reported concerns for their children's future transition into employment, given their challenges in receiving support in school.

…loads of dyslexics that use Access to Work will probably turn around and go actually that was a load rubbish. They gave me a microphone. They gave me a laptop, but where was the actual support when I was having 100 emails and I was getting in trouble because I wasn't answering those emails or, you know, the services aren't necessarily tailored to us… FG26, dyslexic adult

…from the age of 5 to 16, a child is at school… one of the main ways that they are valued is through their academic progress, and I can't help but worry that has to be quite damaging for a person's self‐confidence going into adulthood, going into the workplace… How does a child who has always felt that they are playing catch up with others, how do they make that transition into the workplace? … How could that transition be made easier for them? FG13, parent of dyslexic children

##### 3.2.2.3 Improving Understanding of and Attitudes Towards Dyslexia

Most participants conveyed that there are wide misunderstandings and stigma about dyslexia. They expressed the view that education is not designed for dyslexic people to learn, and there is little recognition of the additional challenges experienced, nor the personal strengths of dyslexic people (first subtheme). Finally, some described how dyslexia is even less understood in people with intersecting identities and the consequences of social inequalities (second subtheme).

##### 3.2.2.4 Recognising and Valuing Strengths and Differences

Many participants expressed the view that the primary format of teaching, involving reading or writing, is a barrier to learning, and consequently, academic attainment and progress do not reflect actual understanding or aptitude. Participants conveyed that there are other skills and ways of learning that the current school system does not understand, which may be limiting attainment.

…he taught himself so much during lockdown. He was using some really complex computer programs that we just were like, well, how did you learn to do that? … He just taught himself. He learns by watching YouTube…, its stuff he wants to do and he wants to learn… I'm a teacher as well… But I can just see how education is just, it's not the one size fits all and there's so many children slipping through the net at the moment and it's really sad. FG21, parent of dyslexic children

Several participants described how there are perceptions that dyslexia is linked to intelligence, and that dyslexic people are stigmatised as ‘thick’ or ‘stupid’, or perceived as ‘lazy’ or ‘naughty’ due to misattributing behaviours stemming from challenges or frustration. Some participants also emphasised the importance that society sees dyslexia as an identity, rather than something that should be cured. A few also noted that these societal views can deter parents and dyslexic people from wanting to be diagnosed and labelled as dyslexic.

…other peoples' perception is quite negative… it's almost, well, she can't be that bright cause she's dyslexic and that is quite demoralizing because you feel like you're battling against a glass ceiling because you're perceived in this way…. But even going into work and in my early career erm there was a tick box that said on the applications, ‘are you dyslexic’, or on certain on‐boarding, and you tick it and people would then know and then it would be said… she's not gonna do that well because she's dyslexic. FG36, dyslexic adult

Numerous participants conveyed that although dyslexia poses challenges, an individual's strengths often go unrecognised. They described their own recognition of the incredible strengths that dyslexic individuals have to offer and that their different ways of thinking could be an asset in many situations.

I think it's amazing how my children spell. I think they're absolutely geniuses… the inventiveness of my daughter amazes me. It's quirky… and I just think life would be pretty boring without it. FG14, parent of dyslexic children

##### 3.2.2.5 Understanding Intersectional Identities and Social Inequalities

Many participants conveyed that they believe there is even less understanding of the presentations of dyslexia, outcomes, and support needs across individuals with co‐occurring conditions (e.g., autism) and intersectional identities, and those who experience social inequalities.

I sometimes feel that maybe that's why I have a kind of atypical presentation because I've got other things that are maybe cancelling some things out in some ways, or augmenting some things in other ways, and that there's this kind of interaction effect there. FG03, dyslexic and autistic adult

Further, a few participants reported how there seems to be a misunderstanding about how dyslexic challenges manifest across different demographics, like genders or ethnicities. They expressed the view that this misunderstanding may mean that dyslexia is missed or that challenges could be attributed to aptitude or other behaviours. Some participants expressed concerns about the impact of social inequalities on outcomes, reflecting on their own/their children's privilege of receiving strong emotional, financial, and academic support from parents and the consequences for children who do not have this.

…looking back that you can very definitely see, you know, the difference between the boys and the girls… the boys, there's that classic tendency of mucking around and being the class comedian, clown, you know or just being really disruptive… because of the frustration that goes with the dyslexia. FG30, dyslexic adult and parent of dyslexic children

The most important support is from their family and if they don't have that, they're sort of left I think to fend for themselves. So, it should be school based…, from my perspective… in a mainstream school, ‘cause there aren't that many specialist schools around. And if you can't afford it or you can't get your kid in, then those opportunities aren't afforded. FG19, parent of dyslexic child

##### 3.2.2.6 Improving Mental Health and Self‐Esteem of Dyslexic People

Many participants conveyed how early negative experiences have consequences for mental health and self‐esteem in dyslexic people. Some dyslexic adults described how feeling like they were failing and the exhaustion of trying to keep up had lasting impacts on their self‐esteem. Also, many parents conveyed that they could see an impact on their child's mental health and self‐esteem due to struggling at school, the stress they are under to achieve, and the negative messaging associated with this.

I had one son who went very rapidly down the behaviour route with exclusions, and one daughter who started school refusing. So very different responses, both entirely from the same position of feeling stupid… self‐esteem bottoming out, hating being different but feeling different all the time… none of their skills being recognized, constantly being criticized, erm, really, really horrible experiences. FG25, parent of dyslexic children

Several participants described feeling the exhausting efforts to camouflage challenges at school and in the workplace. Some dyslexic adults conveyed how they had developed coping strategies to hide their challenges from others at work, but this often meant that they would need to work longer and harder than their colleagues to compensate. Parents also described the pressure for their children to engage with extra tuition and homework to achieve at school, and that this can exacerbate the exhaustion children already feel from having to work harder than their peers.

…it's the exhaustion of having to, you know, a neurotypical person could read an email once and get the grasp of it. I have to read it 3, 4, 5 times. I then have to go away and come back and read it before I send something out. I check it,… I triple check it. FG24, dyslexic adult

She would come home from school from year R and lie on the sofa under a blanket and literally be so tired. I, we couldn't do clubs after school because, she just couldn't… because she was so tired. Whereas all her friends were going off, like all these different things… FG27, parent of dyslexic child

Some participants described their experiences of forming positive self‐identity as a dyslexic person, which helped support self‐esteem, with a diagnosis providing validation and language for the challenges they were experiencing. Some parents also reported that they tried to support their children to see their diagnosis in a positive light, to protect their mental health and self‐esteem. Some participants described positive experiences of relating to other dyslexic people and recognising shared experiences, fostering a sense of community.

…when I got my, like, diagnosis it was just, like the world is kind of aligning for me and it's not something to be embarrassed about, it's something that's kind of given me comfort. FG18, dyslexic adult

It's really nice to know people with dyslexia ‘cause you realize you're not alone and I actually went to the national dyslexic show on Friday and came away high as a kite because everybody there was dyslexic… and it was really empowering actually. FG11, dyslexic adult and parent of dyslexic child

### 3.3 Summary

Community members felt that the current funding landscape is too heavily skewed towards Biology, brain and cognition. Participants conveyed a range of lived experiences relating to important issues in their everyday lives, from which we developed themes relating to priority areas. The community priority research areas are not well captured by the funding focus on Biology, brain and cognition. The first theme (early, effective and accessible identification and diagnosis of dyslexia) fits within the Diagnosis, characteristics and behaviour category. The second theme (effective support for dyslexic people and their families) fits within the Support and interventions category. The third theme (improving understanding of and attitudes towards dyslexia) fits within the Societal issues category. Finally, the fourth theme (improving mental health and self‐esteem) fits within Support and interventions and potentially Services. Our analysis has therefore suggested future priority areas that would require a rebalancing from the predominant focus of previous funding on Biology, brain and cognition. In Study 3, we used these insights from lived experience to develop a survey to quantify priorities over a wider range of participants to obtain ranked priorities for the community.

## 4 Study 3. Survey of the Dyslexia Community

### 4.1 Methods

#### 4.1.1 Survey

We developed a list of research topics for participants to rate on importance. Our initial list reflected the 35 IACC subcategories for funding outlined above, although we removed a few items that were not represented among the funded projects and not mentioned by focus group participants (e.g., immune/metabolic pathways) and collapsed items across subcategories where the distinction was not clear to a lay audience (e.g., epigenetics and genetic risk factors were collapsed to ‘genes’). We reviewed items to ensure that the main themes identified in Study 2 were represented, and accordingly added extra items for mental health and self‐esteem, improving attitudes and understanding of dyslexia, workplace support and development of individualised interventions. We then consulted with five dyslexic individuals who gave feedback that the survey was too long and gave suggestions to improve readability and accessibility. We then rephrased items and collapsed similar items, and following a further iteration of feedback and refinement, agreed on 19 items.

The final survey (https://osf.io/sgy7t/) started with demographics questions to characterise our sample. Participants then rated the importance of the 19 research topics, presented in a random order for each individual, on a 5‐point Likert scale (1 = ‘not at all important’; 2 = ‘not very important’; 3 = ‘moderately important’; 4 = ‘important’; 5 = ‘very important’), with a ‘don't know’ option. Participants then selected their top three research topics and ranked these. The survey ended with optional open‐ended questions about something participants would like to be researched, and something they would not like to be researched, and a required question to provide further views or experiences about dyslexia research (participants were instructed to respond ‘no’ if they had nothing to add).

Survey responses were collected on Survey Monkey. To minimise infiltration by bots, participants were not given a fixed reward but could enter into a draw for 10 £20 Amazon vouchers (Griffin et al. 2022). The draw was not advertised in recruitment materials shared on social media. To help detect bots, we used separate collectors for targeted recruitment and social media, required an open‐text response to detect nonsensical responses (see above), monitored participant completion time, and carefully checked responses with shared IP addresses.

#### 4.1.2 Participants

The same inclusion criteria were used as in Study 2 (Section 3.1.2; e.g., 18+ years, living in the UK). We recruited participants via social media (*n* = 367), The Dyslexia Show, UK (*n* = 82), and targeted emails to schools, dyslexia organisations and research databases (*n* = 109). Overall, 558 participants started the survey. Thirty‐two were excluded for living outside the UK and 87 for not completing all survey items. We removed another participant with a short completion time with nonsensical open‐text responses and a non‐UK IP address, which could reflect a bot, and two dyslexia professionals who were neither dyslexic themselves nor parents/carers or immediate family members of someone with dyslexia. These exclusions resulted in 436 responses (Table 4).

**TABLE 4:** Frequencies of survey respondent demographics.

Response	Whole sample (total *n* = 436)	Dyslexic adults (total *n* = 252)	Parents/carers (total *n* = 302)
Age range			
18–24	13	13	1
25–34	43	40	6
35–44	127	58	96
45–54	168	84	139
55–64	59	37	42
65+	25	20	17
Prefer not to say	1	0	1
Gender			
Female	377	197	280
Male	51	47	21
Other	6	6	0
Prefer not to say	2	2	1
Ethnic group			
White British	388	216	273
White Irish	5	3	2
White—Other	18	12	12
Black African	2	2	2
Black Caribbean	4	4	2
Indian	4	4	3
Pakistani	2	1	2
Asian—Other	1	1	0
Mixed White and Black African	1	1	0
Mixed White and Black Caribbean	2	1	2
Mixed—Other	3	1	2
Other	5	5	1
Prefer not to say	1	1	1
Highest level of education			
Higher degree	177	101	131
Degree	181	99	122
A‐levels/equivalent	38	22	26
GCSEs/equivalent	23	17	12
Vocational/other	13	9	8
No formal qualifications	3	3	3
Prefer not to say	1	1	0
Read or hear about dyslexia research			
Never	77	53	43
Sometimes	239	138	165
Regularly	119	60	93
Prefer not to say	1	1	1
Previous participation in dyslexia research			
Never	349	205	238
Less than once every 2–3 years	40	21	29
Once every 2–3 years	14	7	11
Once a year	17	6	13
More than once a year	14	11	10
Prefer not to say	2	2	1

Of these respondents, 252 reported being dyslexic themselves (207 had a diagnosis, with the remainder self‐identifying), and 302 were a parent/carer to a dyslexic child. One hundred nineteen participants were dyslexic themselves *and* a parent/carer to a dyslexic child. One participant was neither a parent/carer nor dyslexic themselves, but an immediate family member of a dyslexic person. One hundred forty‐nine participants reported additional roles in the dyslexia community (e.g., dyslexia assessors, teachers, tutors and charity involvement). Table 4 shows that most participants were female (86.5%) and White (94.3%), with 82.1% having a degree or higher degree. Parents/carers reported the gender, age, and current schooling (or most recently attended) for each of their children with diagnosed or suspected dyslexia, for up to four children (Table S2). De‐identified survey data are available at https://osf.io/sgy7t/.

#### 4.1.3 Analysis

For each research topic, we calculated mean importance ratings and the percentage of respondents ranking it within their top three. Participants were also asked to rank their top three choices by importance, but we have not analysed this data because it was unclear if participants skipped the question and left the items in the order initially presented, and because we did not unambiguously state that the first position should be used for ‘most important’. C.M. conducted content analysis to identify any topics that participants did (not) want researched that were not captured in the quantitative data. A priori, the main categories were ‘do want researched’ and ‘do not want researched’ with the survey research topics as the subcategories. New subcategories were added inductively where these deductive codes did not fully cover open‐ended responses, which were reviewed before final coding.

### 4.2 Results

#### 4.2.1 Quantitative Analysis

**FIGURE 2:**
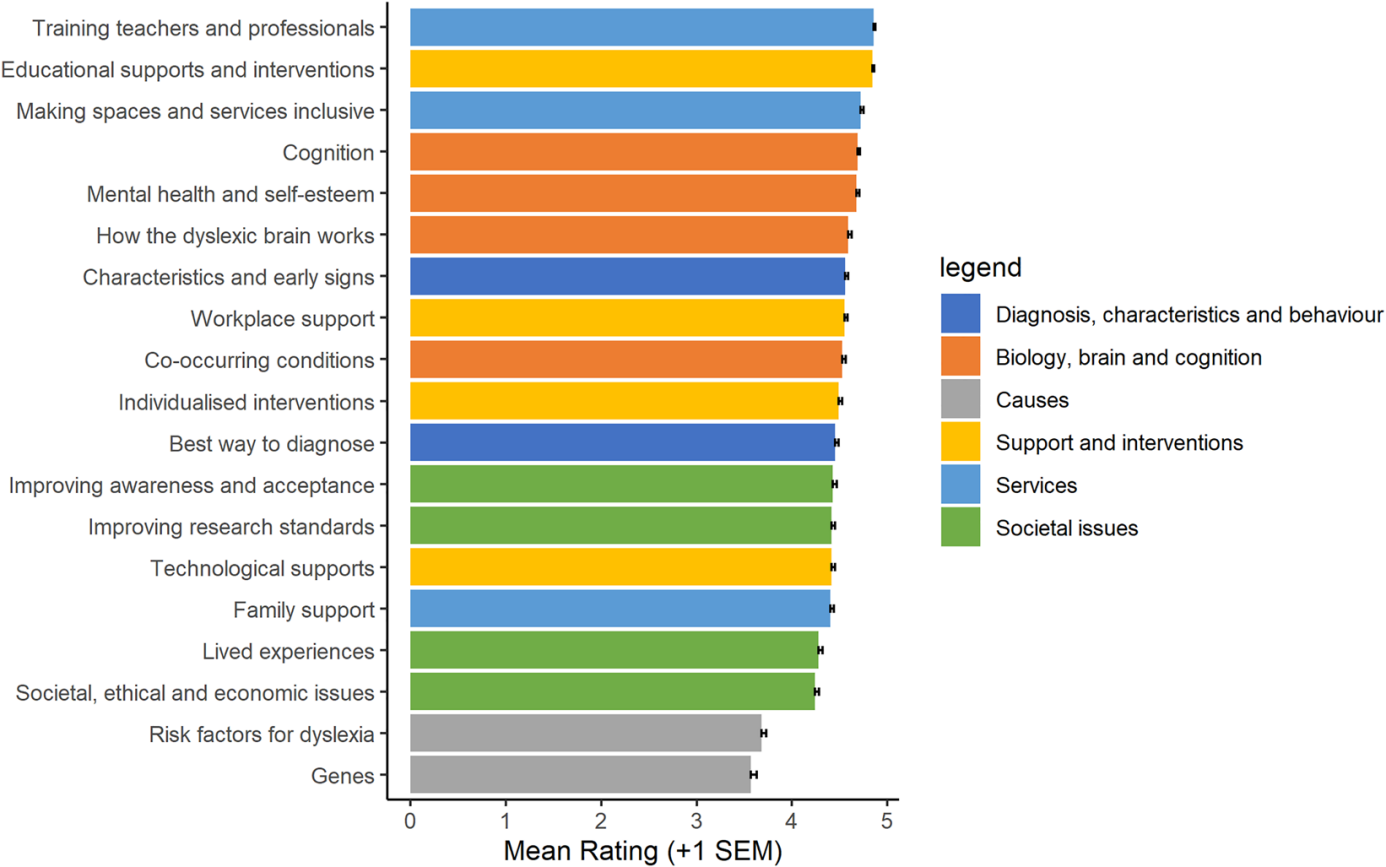
Mean rated importance for each research topic. *Note*. SEM = standard error of the mean. Colours reflect the categories used for funded research projects in Study 1, as in Figure 1. Note that we added items (‘mental health and self‐esteem’, ‘workplace support’, ‘individualised interventions’ and ‘awareness and acceptance’) that were not based on the initial subcategories, but chose the best fitting category for the purposes of this figure. We categorised ‘mental health and self‐esteem’ within the subcategory of ‘co‐occurring conditions’ of ‘Biology, brain and cognition’.

The percentage of ‘don't know’ responses per item ranged from 0% to 1.83% (mean = 0.64%). Figure 2 shows the mean importance ratings for each research topic across all participants, excluding ‘don't know’ responses. All research topics had a mean rating above 3 (*moderately important*). Moreover, all research topics had a mean rating above 4 (*important*), except ‘risk factors for dyslexia’ (*M* = 3.68) and ‘genes’ (*M* = 3.57). The top five highest ratings were given for ‘training teachers and professionals’ (*M* = 4.85), ‘educational supports and interventions’ (*M* = 4.84), ‘making spaces and services inclusive’ (*M* = 4.72), ‘how do people with dyslexia think and process information’ (i.e., cognition; *M* = 4.69) and ‘mental health and self‐esteem’ (*M* = 4.68). When looking at responses from dyslexic adults and parents/carers of those with dyslexia separately, we found a similar pattern of results, with the same top five highest‐rated research topics (Figure S1).

**FIGURE 3:**
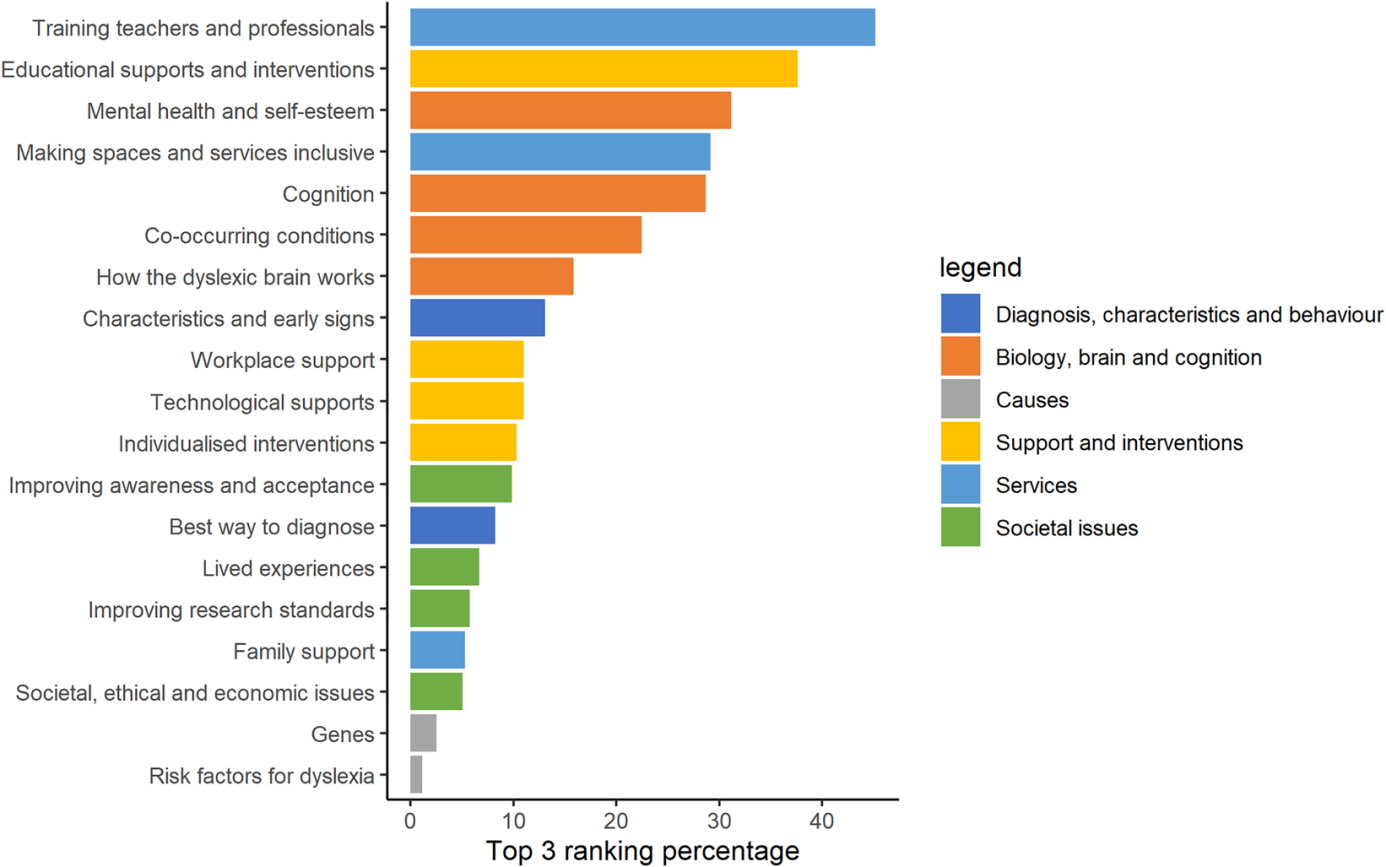
Percentage of participants who selected each research topic within their top three ranking.

The same top five research topics emerged in the percentage of participants selecting each research topic in their ‘top 3’, both across the whole sample (Figure 3) and in dyslexic adults and parents/carers (Figure S2). The exact ordering of these top five research topics varied subtly for dyslexic adults and parents/carers. For example, ‘educational supports and interventions’ was more often selected by parents/carers in their top three (44.7%) than for dyslexic adults (28.6%). However, training teachers and professionals was the most commonly chosen item for both dyslexic adults (38.1%), and parents/carers of those with dyslexia (52.3%) and ‘genes’ and ‘risk factors for dyslexia’ were the least likely to be selected for both dyslexic adults (3.6% and 1.6%, respectively, along with family support at 3.6%) and parents/carers (2.0 and 0.33%, respectively).

#### 4.2.2 Qualitative Analysis

Full content analysis results are presented in Tables S3 and S4. In terms of research topics not fully captured by our survey with ≥ 10 references, 24 people wanted future research into the strengths and benefits associated with dyslexia, 14 wanted research about exam and test accommodations, 12 wanted research into the effects of timing of diagnosis and support and 10 wanted research into dyslexia in adulthood, including links to ageing and dementia. The topics that participants did not want to be researched were fewer and more varied, although six participants wrote that they did not want to see future research designed to identify deficits in dyslexic people.

### 4.3 Summary

Overall, all research topics in our survey were perceived to be relatively important. A consistent top five emerged from the survey results, both when looking at mean ratings and percentage of top three rankings, and for both dyslexic adults and parents/carers of dyslexic children. Identifying the best ways to train teachers and professionals was consistently identified as the top research priority, which chimes with themes from our qualitative focus group analysis (‘early, effective and accessible identification and diagnosis’ and ‘improving understanding of and attitudes towards dyslexia’). ‘Educational supports and interventions’ and ‘mental health and self‐esteem’ were also within the top five, mapping onto other themes from our focus group analysis. While the item for improving understanding and acceptance of dyslexia, which we added following our focus group analysis, was not in the top five, making spaces and services inclusive was in the top five, which is a related concept. Perhaps surprisingly on the basis of our focus group analysis, cognition (“how do people with dyslexia think and process information”) was also in the top five. This is positive given that a relatively high proportion of previous funding (Study 1) has been allocated to cognition projects. The other top five topics, meanwhile, have received relatively little funding to date.

Content analysis allowed us to identify further topics that participants wanted future research to focus on, including the strengths and benefits associated with dyslexia, which also relates to a subtheme under ‘Improving understanding of and attitudes towards dyslexia’ from our focus group analysis. Other desired topics included research into exam and test accommodations, and research into lifespan issues, including adulthood and ageing, and the impact of early or late diagnosis. Some of these topics were also represented in the focus group themes, but we had not been able to include survey items for each of these topics without the survey becoming too long. However, future surveys could be developed to ask participants to rank these items, to establish their priority relative to other areas.

## 5 General Discussion

Our funding analysis showed that the majority of UK grant expenditure for dyslexia research has been awarded to Biology, brain, and cognition projects. While some community members in our focus groups saw value in this research area, their overall feeling was that more funding needs to be invested in research areas that are more directly relevant to dyslexic people's everyday lives. We developed four important areas for future research from the lived experiences and perspectives of focus group participants: (1) early, effective and accessible identification and diagnosis of dyslexia; (2) effective support for dyslexic people and their families; (3) improving understanding of and attitudes towards dyslexia and (4) improving mental health and self‐esteem of dyslexic people. These themes are inter‐linked. For example, participants conveyed how diagnosis was important for accessing support (c.f. Colenbrander et al. 2018), attitudes towards dyslexia can be linked to self‐esteem (c.f. Gibby‐Leversuch et al. 2021; Livingston et al. 2018), and better understanding of different presentations of dyslexia could in theory lead to better diagnosis (c.f. Catts et al. 2024).

We used these insights to inform our survey which was completed by a wider range of participants to develop quantitative data relating to the community's top priorities. All research topics, including ‘Biology, brain and cognition’ topics, were rated as moderately to very important. Overlapping with insights from the focus groups, the priority research areas related to (1) training teachers and professionals, (2) educational supports and interventions, (3) mental health and self‐esteem, (4) making services and spaces more inclusive and (5) cognition. Open‐ended responses highlighted additional focuses for future research, including research into the strengths and benefits associated with dyslexia, impact of early/late diagnosis, adulthood and exam and test accommodations—some of which had also been discussed in focus groups.

Many of our findings align with research into other conditions. For example, the predominance of funding awarded to basic science research relative to applied research has been reported for autism research (den Houting and Pellicano 2019; Krahn and Fenton 2012; Pellicano et al. 2013; Singh et al. 2009). Dissatisfaction from community members regarding this funding allocation has also been reported for autism and genetic syndromes research (Cristescu et al. 2024; Pellicano et al. 2014) and has begun to change how research is conducted (Fletcher‐Watson et al. 2021). Other research priority setting exercises have identified similar priorities. For example, Lim et al.'s (2019) top research priority for children and young people with learning difficulties, which was verified as uncertainty from prior research evidence, was about identifying the knowledge, skills, and training needed by educational professionals to detect and support children with learning difficulties, which echoes our themes from focus groups (early, effective and accessible identification and diagnosis of dyslexia; effective support for dyslexic people and their families), and the highest prioritised research topic in our survey (training teachers and professionals). Most of Lim et al.'s other research priorities overlap with the topics prioritised in our study, although there are some points of difference. For example, the role of health, social work, and ‘third sector’ was not discussed by our participants, but was in Lim et al., and mental health and self‐esteem were not explicitly mentioned in Lim et al.'s top priorities. These differences may reflect the greater range of conditions included in Lim et al.'s study, their focus on children and young people, and research methodology. These differences also highlight the importance of consulting specific communities.

Following the focus group findings, which suggested that ‘Biology, brain and cognition’ research had been over‐emphasised and poor value for money, we were surprised that cognition (“how do people with dyslexia think and process information”) appeared in the top five prioritised items from the survey. This could be because survey participants' judgements were not influenced by the funding data, but also due to differences in how ‘cognition’ was described in the survey compared to the focus groups. It is also possible that the grouping of ‘Biology, brain and cognition’ led focus group participants to focus on ‘Biology’ and ‘Brain’, without fully communicating their views into ‘Cognition’ specifically. Accordingly, most of the references in content analysis referred to biological and brain‐based research rather than cognition. It may therefore be useful to separate cognition from biology and brain in future investigations, as in a recent priority setting exercise for genetic syndromes (Cristescu et al. 2024).

One important caveat to the results presented here and in other funding portfolio analyses is that the different research categories are not completely distinct. This caveat may be particularly important for dyslexia research, where cognition overlaps with diagnostic tests (e.g., reading tests). Biology, brain and cognition are inherently linked to Causes. In turn, Support and interventions and Diagnosis are also linked to these categories. As an example, phonological processing difficulties are a cognitive factor (with neurobiological underpinnings; Ramus 2004) that have a causal role in dyslexia (Pennington 2006; Share 2021; Snowling 1998, for reviews), and this has resulted in phonological‐based interventions (Castles et al. 2018, for review) and screening tools (Duff et al. 2015). Categorising funded research projects into distinct categories was therefore not always straightforward, but it did provide a useful stimulus for eliciting participant views.

Our funding portfolio analysis has shown which research projects have been funded, which is determined by both what researchers want to research (and/or what they think has a chance of being funded) and what funders decide to fund. To understand the relative importance of these factors, it would be interesting to characterise unfunded research by categorising publications, to see whether the same emphasis on Biology, brain and cognition is evident. It would also be informative to understand the proportion of funding applications made relative to the awards made in each research category, although this is difficult as unfunded applications are not publicly available.

Our work has focused on identifying community perspectives about past and future dyslexia research. This differs from James Lind Alliance partnerships (e.g., Lim et al. 2019; Morris et al. 2015) which work collaboratively with researchers to identify under‐researched priority areas. As most of our participants were not regular consumers of dyslexia research, some of the research questions that participants raised may have already been studied. For example, a few survey respondents said they wanted to know whether dyslexia is hereditary or not, which has been long established (Pennington 1989). As we expected, some focus group participants mentioned having difficulty formulating specific research questions, which is why our RTA focused on drawing out broad themes of importance to participants, which researchers can then use to guide the development of specific research questions. There is a question about whether the priority areas identified by participants are best targeted by research, or if participants are really asking for more funding to be spent on these practical issues (i.e., do participants really want more research into how to train professionals, or do they just want to see generally more funding directed to training professionals?). However, it is our view that research has the potential to tackle these challenges, for example, by asking what are the most effective ways of training teachers given the constraints on funding and time, or developing new training programmes to help target teachers' misconceptions about dyslexia (Peltier et al. 2022). We believe that foregrounding the lived experiences and important issues for dyslexic people will help researchers understand the very real, everyday challenges that research can solve to improve dyslexic people's lives. Working with dyslexic people and their families to devise new research studies will be important to ensure that they are well placed to provide translational benefits.

Another implication arising from our research is that the dissemination of dyslexia research needs improving. Several focus group participants commented that dyslexia research is not accessible or clearly translated to the community. If researchers believe that the research priorities raised here have already been addressed, then instead of conducting new research, the most important next steps are to summarise the results of existing studies and present them in accessible formats for wide dissemination, outside of academic journals. The participant views captured in our study are likely influenced by understandings of the research aims of different types of research and how basic science can lead to translational benefit. Future research could therefore target specific research areas to get deeper insights into participant understandings and perceptions, and investigate how this influences their priorities. Ultimately, researchers focusing on Biology, brain and cognition may need to clearly communicate the value of their research to community members, including emphasising where findings have been translated into support and interventions (e.g., research into phonological processing, Castles et al. 2018).

Although we strived to recruit diverse samples by working with charities and including undiagnosed participants, we note that our samples are not representative of the population. According to Census data (Education, England and Wales—Office for National Statistics 2021), 33.8% of residents aged ≥ 16 years in England and Wales had a degree (or equivalent) or higher qualification, whereas this percentage was 78.4% and 82.1% for our focus group and survey participants, respectively, showing that our participants were overall very highly educated. Similarly, 82% of people identified with a White ethnic group in the Census (Barton 2024), compared to 86.4% and 94.3% in our focus group and survey participants, respectively. Our participants were also mostly female, despite males being more likely to be dyslexic (Knight and Crick 2021; Miles et al. 1998; Rutter et al. 2004). While some disproportionality of demographics might be expected (Knight and Crick 2021; Strand and Lindorff 2021; Strand and Lindsay 2009), future research is needed to determine the generalisability of our findings across the entire dyslexia community and to investigate the role of intersectionality, especially as understanding intersectionality was a subtheme from Study 2. By achieving more diverse representation, we will be able to ensure that research priorities are not biased towards the views of a small subsection of society. Future research could also use tailored methods to capture the perspectives of dyslexic children (Modanloo et al. 2024; Postma et al. 2022).

Overall, dyslexia community members wanted to see more research investment in areas of direct, practical relevance to their everyday lives. The UK's total investment in dyslexia research is relatively low (approximately £15.9 million over 22 years, compared to £20.8 million over 5 years for autism research; Pellicano et al. 2013), and no research topics were deemed ‘not important’ by the community. We would therefore not recommend that funding be *reduced* from any research areas (particularly in light of basic science having led to evidence‐based practices in dyslexia; e.g., Castles et al. 2018), but instead that funding be *increased* for under‐funded areas. Indeed, it is unlikely that money would simply be re‐allocated from one category to another, as the different research categories lend themselves to different funder remits and therefore different pots of money. Therefore, when allocating funding, it is often less about competition between these research categories as opposed to competition between dyslexia research and non‐dyslexia research.

We hope that this paper will help researchers to identify research questions that will bring benefit to dyslexic people and their families, and that these priorities will help to strengthen the case to suitable funders, who are increasingly committed to incorporating community perspectives into funding decisions (e.g., NIHR 2015). Importantly, by involving dyslexic people and their families in all dyslexia research projects and funding decisions, we will be able to bridge the gap between funding allocation and community relevance. While this investigation is focused on the UK context, it has been noted that community involvement in research might be further ahead in the UK than in other countries (Pratt 2021). We therefore would expect similar gaps to exist between dyslexia research funding and community priorities in other countries, which is an important focus for future research.

## Conflicts of Interest

C.M. and H.J. are active dyslexia researchers who are eligible for, and have applied for and/or received, research funding from some of the funding sources reported. The other authors declare no conflicts of interest.

## Supporting information


**Data S1.** Supporting Information.

## Data Availability

The data and materials that support the quantitative findings of this study are openly available in the Open Science Framework at https://osf.io/sgy7t/. Transcripts of qualitative data are not shared due to privacy concerns, but we share materials (e.g., scripts).
