# Supplementary material for: What Are the Research Priorities for the Dyslexia Community in the United Kingdom and How Do They Align With Previous Research Funding?
Source: Dyslexia. 2025 Mar 18;31(2):e70004. doi: 10.1002/dys.70004 (PMC11933878; doi:10.1002/dys.70004)

**Supplementary Materials**

**Supplementary Table 1.**

*Number of Projects and Amount Awarded for each of the Subcategories in the Biology, Brain and Cognition category*

| Subcategory | | N projects | Amount awarded (£) |
| --- | --- | --- | --- |
| ii.6 | Cognitive studies | 16 | 2,357,696 |
| ii.7 | Computational science | 2 | 441,568 |
| ii.8 | Co-occurring conditions | 1 | 278,700 |
| ii.9 | Developmental trajectory | 3 | 1,452,841 |
| ii.10 | Immune/metabolic pathways | 0 | 0 |
| ii.11 | Molecular pathways | 1 | 1,393,036 |
| ii.12 | Neural systems | 4 | 2,598,646 |
| ii.13 | Neuropathology | 0 | 0 |
| ii.14 | Sensory and motor function | 5 | 3,699,944 |
| ii.15 | Subgroups/biosignatures | 0 | 0 |
| ii.16 | Model systems | 1 | 211,787 |

**Supplementary Table 2.**

*Frequencies of Reported Characteristics for Children With Diagnosed or Suspected Dyslexia*

| Response | Frequency |
| --- | --- |
| Number of Children With Dyslexia Per Respondent (total n = 302) | |
| 1 | 203 |
| 2 | 86 |
| 3 | 7 |
| 4+ | 5 |
| Prefer not to say | 1 |
| Gender of Children With Dyslexia (total n = 416) | |
| Female | 226 |
| Male | 186 |
| Other | 1 |
| Prefer not to say | 3 |
| Current Or Most Recent Schooling of Children With Dyslexia (total n = 416) | |
| State mainstream school | 257 |
| State special school | 1 |
| Private mainstream school | 76 |
| Private special school | 19 |
| Not attending school (under 5) | 1 |
| Not attending school (home schooled) | 2 |
| Not attending school (other) | 7 |
| Other | 51 |
| Prefer not to say | 2 |
| Age of Children With Dyslexia (total n = 416) | |
| 4 – 10 years | 226 |
| 11 – 17 years | 119 |
| 18+ years | 71 |

*Note.* Parents/carers were asked to report their children’s most recently attended schooling if they had left school.

**Supplementary Table 3.**

*Results of Content Analysis of Open-ended Survey Responses to One Thing Participants Would Like to See Researched in Future.*

| Subcategory | N references |
| --- | --- |
| Do Want Researched – Survey Items | |
| Best way to diagnose | 17 |
| Brain | 8 |
| Characteristics and early signs | 7 |
| Co-occurring conditions | 16 |
| Cognition | 8 |
| Educational supports and interventions | 49 |
| Family support | 1 |
| Genes | 6 |
| Improving awareness and acceptance | 9 |
| Improving research standards | 1 |
| Individualised interventions | 7 |
| Lived experiences | 1 |
| Making spaces and services more inclusive | 14 |
| Mental health and self-esteem | 19 |
| Risk Factors | 7 |
| Societal, ethical and economic issues | 5 |
| Technological interventions | 3 |
| Training teachers and professionals | 17 |
| Workplace support | 6 |
| Do Want Researched – Other | |
| Adults and ageing | 10 |
| Careers advice | 1 |
| Compensatory strategies | 1 |
| Crime and criminal justice | 4 |
| Cure | 1 |
| Does dyslexia exist | 1 |
| Effect of timing of diagnosis and support | 12 |
| Effect on sleep | 1 |
| Exam and test adjustments | 14 |
| Healthcare professionals | 1 |
| Impacts on relationships and social skills | 4 |
| Intersectionality | 4 |
| Living with dyslexia | 1 |
| Neurodiversity | 8 |
| Non-educational outcomes | 1 |
| Organisation skills | 2 |
| Sex/gender differences | 4 |
| Strengths | 24 |
| Support resources | 1 |
| Support with written instructions | 2 |
| Visual interventions and supports | 3 |

**Supplementary Table 4.**

*Results of Content Analysis of Open-ended Survey Responses to One Thing Participants Would Not Like to See Researched in Future.*

| Subcategory | N participants |
| --- | --- |
| Do Not Want Researched – Survey Items | |
| Best way to diagnose | 1 |
| Brain | 1 |
| Characteristics and early signs | 2 |
| Co-occurring conditions | 0 |
| Cognition | 0 |
| Educational supports and interventions | 4 |
| Family support | 0 |
| Genes | 19 |
| Improving awareness and acceptance | 1 |
| Improving research standards | 0 |
| Individualised interventions | 1 |
| Lived experiences | 1 |
| Making spaces and services more inclusive | 0 |
| Mental health and self-esteem | 0 |
| Risk Factors | 6 |
| Societal, ethical and economic issues | 0 |
| Technological interventions | 2 |
| Training teachers and professionals | 1 |
| Workplace support | 0 |
| Do Not Want Researched – Other | |
| Animal research | 1 |
| Cure | 1 |
| Deficits | 6 |
| Does dyslexia exist | 1 |
| Embryo research | 1 |
| Intersectionality | 1 |
| Living with dyslexia | 1 |
| Masking | 1 |
| Social exclusion and marginalisation | 1 |
| Visual dyslexia and supports | 2 |

Note. A few responses suggested that participants were responding as a continuation of one thing they *would* like to be researched, but this was not fully clear. To avoid researcher bias, we still analysed these responses. As this reflected a minority of responses, it has not affected the overall conclusions made.

**Supplementary Figure 1.**

*Mean Rated Importance for each Research Topic for Dyslexic Adults and Parents/Carers of those with Dyslexia*


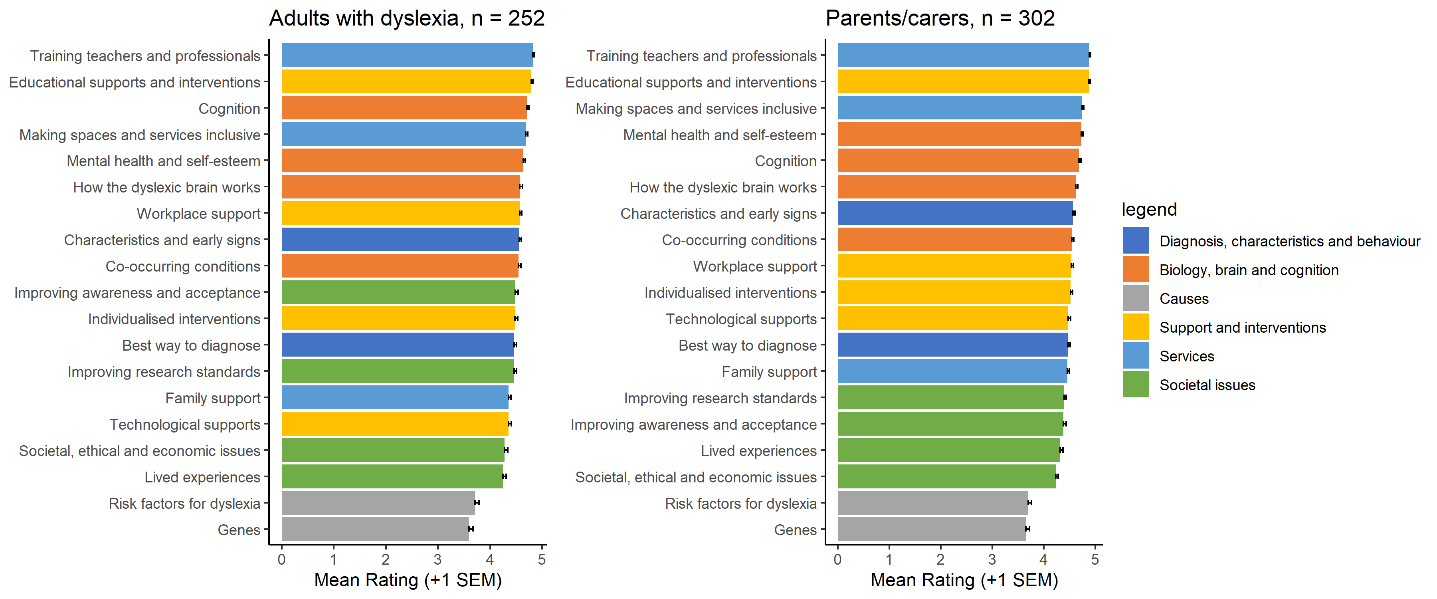


**Supplementary Figure 2.**

*Percentage of Adults with Dyslexia and Parents/Carers of those with Dyslexia who Selected Each Research Topic within their Top 3 Ranking*


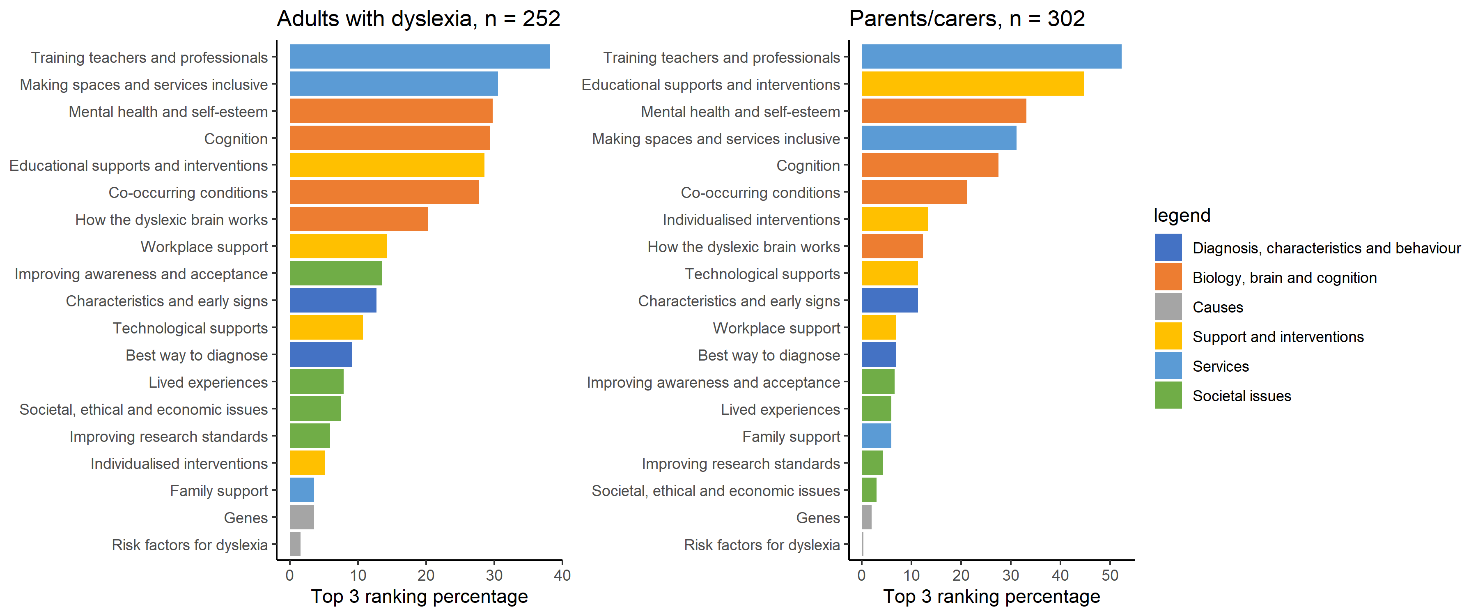

Supplement: Supplementary file 1 — Data S1. Supporting Information. [file DYS-31-e70004-s001.docx]
